# Circulating Zonulin, a Marker of Intestinal Permeability, Is Increased in Association with Obesity-Associated Insulin Resistance

**DOI:** 10.1371/journal.pone.0037160

**Published:** 2012-05-18

**Authors:** José María Moreno-Navarrete, Mònica Sabater, Francisco Ortega, Wifredo Ricart, José Manuel Fernández-Real

**Affiliations:** Department of Diabetes, Endocrinology and Nutrition, Institut d’Investigació Biomèdica de Girona (IdIBGi), CIBEROBN (CB06/03/010) and Instituto de Salud Carlos III (ISCIII), Girona, Spain; University of Tor Vergata, Italy

## Abstract

Zonulin is the only physiological mediator known to regulate intestinal permeability reversibly by modulating intercellular tight junctions. To investigate the relationship between intestinal permeability and obesity-associated metabolic disturbances in humans, we aimed to study circulating zonulin according to obesity and insulin resistance. Circulating zonulin (ELISA) was measured in 123 caucasian men in association with inflammatory and metabolic parameters (including minimal model-measured insulin sensitivity). Circulating zonulin increased with body mass index (BMI), waist to hip ratio (WHR), fasting insulin, fasting triglycerides, uric acid and IL-6, and negatively correlated with HDL-cholesterol and insulin sensitivity. In multiple regression analysis, insulin sensitivity (p = 0.002) contributed independently to circulating zonulin variance, after controlling for the effects of BMI, fasting triglycerides and age. When circulating IL-6 was added to this model, only BMI (p = 0.01) contributed independently to circulating zonulin variance. In conclusion, the relationship between insulin sensitivity and circulating zonulin might be mediated through the obesity-related circulating IL-6 increase.

## Introduction

Obesity has been associated with increased intestinal permeability and absorption [1]. Obesity is correlated with dramatic increases in intestinal absorptive capacity by increasing in amounts of absorptive mucosa [1]. In mice, it has been reported that intestinal weight, length, and mucosal mass increased significantly with diabetes [2].

Intestinal permeability regulates molecular trafficking between the intestinal lumen and the submucosa, leading to either tolerance or immunity to non–self-antigens [3], [4]. The intercellular tight junctions (TJs) tightly regulate this paracellular antigen trafficking. TJs are appreciated to be extremely dynamic structures operative in several key functions of the intestinal epithelium under both physiological and pathological circumstances [5]. Persistent high circulating levels of inflammatory cytokines, which are often observed in obese subjects, may be an important contributor to intestinal barrier dysfunction by altering structure and localization of TJs [6].

Zonulin is the only physiological mediator known to regulate intestinal permeability reversibly by modulating intercellular TJs [7], [8]. Human zonulin is a ≈47-kDa protein that increases intestinal permeability in small intestine and participates in intestinal innate immunity. Circulating zonulin in serum is considered as a useful marker of intestinal permeability [8], [9]. In fact, in humans it has been validated using lactulose/manitol tests, being serum zonulin strongly correlated with the lactulose/manitol ratio (or intestinal permeability) [10]. Lactulose/mannitol (La/Ma) test is currently used in the investigation of intestinal permeability in several gastrointestinal diseases and malnutrition. This test is performed by oral administration of two sugar probes followed by the determination of their amounts excreted in the urine over a 5-h period [11].”

To gain insight in the relationship between intestinal permeability and obesity-associated metabolic disturbances in humans, we hypothesized a possible association between circulating zonulin, obesity and insulin sensitivity according to glucose tolerance.

**Table 1:** Anthropometrical and biochemical parameters from the participants of the study.

	NGT	GI	P
**N**	82	41	
**Age (years)**	48.29±11.7	55.9±10.3	**0.001**
**BMI (Kg/m^2^)**	26.6±3.2	28.1±3.8	**0.03**
**WHR**	0.929±0.064	0.952±0.069	0.06
**Fasting glucose (mg/dl)**	92.9±7.3	101.15±10.47	**<0.0001**
**Fasting insulin (mg/dl)**	8.1±3.6	11.1±6.5	0.01
**HbA1c (%)**	4.78±0.32	4.9±0.42	0.1
**Log IS (10^−4^/min^−1^·mU** **per liter)**	0.58±0.2	0.43±0.16	**<0.0001**
**HDL-Cholesterol (mg/dl)**	52.8±12.9	52.2±10.1	0.8
**Fasting triglycerides (mg/dl)**	83 (58.5–120.5)	100 (69.5–129)	0.5
**Uric acid (mg/dl)**	5.63±1.3	6.12±1.4	0.05
**Circulating IL-6 (pg/ml)**	1.22±1.1	1.37±0.9	0.4
**Zonulin (ng/ml)**	9.1±4.5	10.9±4.8	**0.03**

NGT, participants with normal glucose tolerance; GI, participants with glucose intolerance; WHR, waist to hip ratio; HbA1c, Glycosylated haemoglobin; IS, insulin sensitivity.

**Figure 1:**
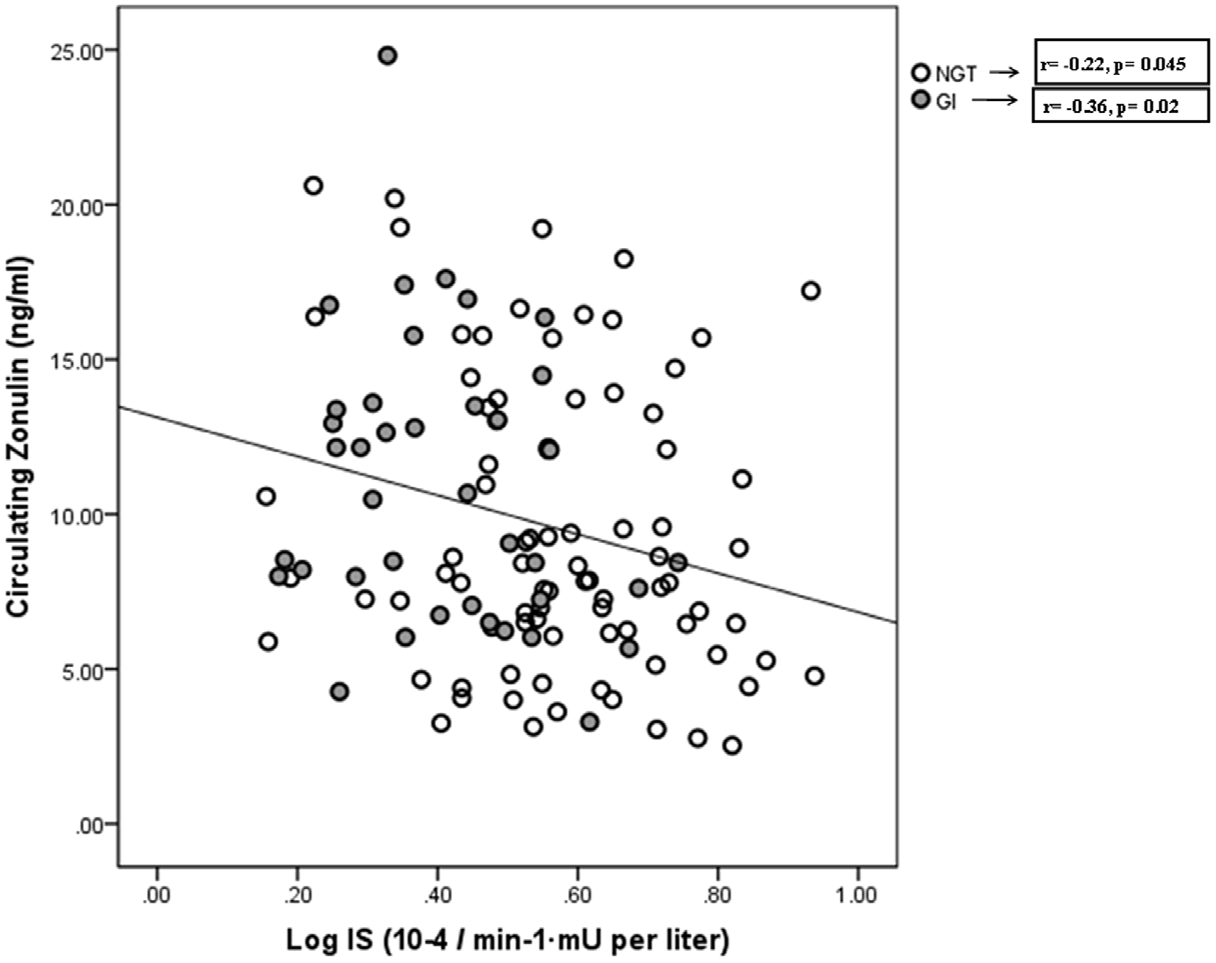
The correlation between insulin sensitivity and circulating zonulin in participants with normal glucose tolerance (NGT, n = 82) and with glucose intolerance (GI, n = 41).

## Methods

### Participants’ Recruitment and Anthropometric Measurements

One hundred twenty three Caucasian men were recruited and studied in ongoing study dealing on non-classical cardiovascular risk factors in Northern Spain. Subjects were randomly localized from a census and they were invited to participate. The participation rate was 71%. A 75 g oral glucose tolerance test according to the American Diabetes Association Criteria was performed in all subjects. All subjects with normal glucose tolerance (n = 82) had fasting plasma glucose <7.0 mM and two-hour post-load plasma glucose <7.8 mM after a 75 g oral glucose tolerance test. Glucose intolerance was diagnosed in 41 subjects according to the American Diabetes Association Criteria (post-load glucose between 7.8 and 11.1 mmol/l). Inclusion criteria included the following: 1) BMI <40 kg/m^2^, 2) absence of systemic disease, and 3) absence of infection within the previous month. None of the control subjects were under medication or had evidence of metabolic disease other than obesity. Alcohol and caffeine were withheld within 12 h of performing the insulin sensitivity test. Liver disease and thyroid dysfunction were specifically excluded by biochemical work-up. All subjects gave written informed consent after the purpose of the study was explained to them. The ethical committee of the Hospital Universitari Dr. Josep Trueta (Comitè d’Ètica d’Investigació Clínica, CEIC) approved the protocol.

Subjects were studied in the post-absorptive state. BMI was calculated as weight (in kilograms) divided by height (in meters) squared. Subjects’ waists were measured with a soft tape midway between the lowest rib and the iliac crest; hip circumference was measured at the widest part of the gluteal region; and waist-to-hip ratio (WHR) was accordingly calculated.

**Table 2:** Pearson’s correlation among circulating zonulin and metabolic parameter.

	All participants(N = 123)		NGT(N = 82)		GI(N = 41)	
	r	p	r	p	r	p
**Age (years)**	0.18	0.05	0.22	**0.045**	−0.03	0.8
**BMI (Kg/m^2^)**	0.28	**0.002**	0.16	0.1	0.42	**0.007**
**WHR**	0.2	**0.025**	0.18	0.1	0.17	0.2
**Fasting glucose (mg/dl)**	−0.01	0.9	−0.03	0.7	−0.11	0.4
**Fasting insulin (mg/dl)**	0.37	**<0.0001**	0.18	0.1	0.57	**<0.0001**
**HbA1c (%)**	0.14	0.1	0.24	**0.03**	−0.04	0.8
**Log IS (10^−4^/min^−1^·mU** **per liter)**	−0.28	**0.002**	−0.22	**0.045**	−0.36	**0.02**
**HDL-Cholesterol (mg/dl)**	−0.21	**0.02**	−0.27	**0.01**	−0.04	0.8
**Log Fasting triglycerides** **(mg/dl)**	0.21	**0.02**	0.22	**0.045**	0.15	0.35
**Uric acid (mg/dl)**	0.2	**0.025**	0.24	**0.03**	0.01	0.9
**Circulating IL-6 (pg/ml)**	0.29	**0.008**	0.31	**0.01**	0.22	0.2

NGT, participants with normal glucose tolerance; GI, participants with glucose intolerance; WHR, waist to hip ratio; HbA1c, Glycosylated haemoglobin; IS, insulin sensitivity.

### Study of Insulin Sensitivity

Insulin sensitivity was measured using the frequently sampled intravenous glucose tolerance test (FSIVGTT). In brief, basal blood samples were drawn at –15 and –5 min, after which glucose (300 mg/kg body wt) was injected over 1 min starting at time 0. At 20 min, regular insulin (Actrapid, Novo, Denmark; 0.03 U/kg) was injected as a bolus. Additional samples were obtained from a contralateral antecubital vein at times 1, 2, 3, 4, 5, 6, 7, 8, 10, 12, 14, 16, 19, 20, 22, 23, 24, 25, 27, 30, 40, 50, 60,70, 80, 90, 100, 120, 140, 160, and 180 min. Samples were rapidly collected via a three-way stopcock connected to the butterfly needle. Data from the FSIVGTT were submitted to computer programs that calculate the characteristic metabolic parameters by fitting glucose and insulin to the minimal model that describes the times course of glucose and insulin concentrations. The glucose disappearance model, by accounting for the effect of insulin and glucose on glucose disappearance, provides the parameters S_I_ (10^−4^) per minute per microunit per milliliter) or the insulin sensitivity index, a measure of the effect of insulin concentrations above the basal level to enhance glucose disappearance. The estimation of model parameters was performed according to the MINMOD computer program [12].

**Table 3:** Multiple linear regression analysis with circulating zonulin as dependent variable.

Model 1	Beta (Standardized coefficients)	T	p
**Age (years)**	0.157	1.77	0.08
**BMI (Kg/m^2^)**	0.066	0.59	0.5
**Log IS (10^−4^/min^−1^·mU per liter)**	−0.263	−2.95	**0.004**
**Log Fasting triglycerides (mg/dl)**	0.132	1.45	0.15
**Model 2**			
**Age (years)**	0.134	1.16	0.25
**BMI (Kg/m^2^)**	0.159	1.34	0.18
**Log IS (10−4/min−1·mU per liter)**	−0.150	−1.29	0.20
**Log Fasting triglycerides (mg/dl)**	0.067	0.579	0.50
**Circulating IL-6 (pg/ml)**	0.23	2.03	0.04

### Analytical Methods

Serum glucose concentrations were measured in duplicate by the glucose oxidase method using a Beckman glucose analyzer II (Beckman Instruments, Brea, California). Glycosylated haemoglobin **(**HbA1c) was measured by the high-performance liquid chromatography method (Bio-Rad, Muenchen, Germany, and autoanalyser Jokoh HS-10, respectively). Intraassay and interassay coefficients of variation were less than 4% for all these tests. Serum insulin was measured in duplicate by monoclonal immunoradiometric assay (Medgenix Diagnostics, Fleunes, Belgium). The intra-assay coefficient of variation was 5.2% at a concentration of 10 mU/l and 3.4% at 130 mU/l. The interassay coefficients of variation were 6.9 and 4.5% at 14 and 89 mU/l, respectively. Total serum triglycerides were measured through the reaction of glycerol-phosphate-oxidase and peroxidase on a Hitachi 917 instrument (Roche, Mannheim, Germany). HDL cholesterol was quantified after precipitation with polyethylene glycol at room temperature. Total serum triglycerides were measured through the reaction of glycerol/phosphate/oxidase and peroxidase. Uric acid was determined by routine laboratory tests. Serum zonulin concentrations were measured by zonulin ELISA Kit (K5600, Immundiagnostik AG, Bensheim, Germany). Intra- and interassay coefficients of variation for these determinations were between 3–7% and between 5–12%, respectively. The ELISA kit used for zonulin measurement only detects the active (uncleaved) form of zonulin. Serum IL-6 concentrations were measured using a solid-phase, enzyme-labeled, chemiluminescent sequential immunometric assay (Immulite 2000; DPC DIPESA S.A., Madrid, Spain). Analytical intra-assay sensitivity was 0.5 pg/ml. No cross-reactivity with other cytokines was evident. Serum samples were diluted and assayed according to the manufacturer’s instructions. Intra- and interassay coefficients of variation for IL-6 determinations were between 5–10%.

### Statistical Analysis

Statistical analyses were performed using SPSS 12.0 software. Unless otherwise stated, descriptive results of continuous variables are expressed as mean and SD for Gaussian variables. Parameters that did not fulfill normal distribution were mathematically transformed to improve symmetry for subsequent analyses. The relation between variables was analyzed by simple correlation (Pearson’s test) and multiple regression analyses. Unpaired t tests were used to compare subjects with NGT and AGT subjects. Levels of statistical significance were set at *P*<0.05.

## Results

In all subjects, as a whole circulating zonulin was significantly increased in obese (n = 33) versus non-obese (n = 90) subjects (12.5±4.6 vs. 9.3±5.1, p = 0.007) and in subjects with glucose intolerance (10.9±4.8 vs. 9.1±4.5, p = 0.03) (Table 1). Circulating zonulin increased with body mass index (BMI), waist to hip ratio (WHR), fasting insulin, fasting triglycerides, uric acid and IL-6, and was negatively associated with HDL-cholesterol and insulin sensitivity (Figure 1) (Table 2).

The associations of circulating zonulin with metabolic variables according to glucose tolerance are described in Table 2.

In multiple regression analysis, insulin sensitivity (p = 0.002) contributed independently to circulating zonulin variance, after controlling for the effects of BMI, fasting triglycerides and age (Table 3). When circulating IL-6 was added to this model, only BMI (p = 0.01) contributed independently to circulating zonulin variance (Table 3).

## Discussion

To the best of our knowledge, this is the first study that associates circulating zonulin concentration (a marker and modulator of intestinal permeability [7]–[10]) with obesity and insulin resistance. Circulating zonulin concentration was also associated with obesity-related metabolic disturbances. After performing multiple linear regression analysis, insulin sensitivity was the main contributor to circulating zonulin variance, but this association was no longer significant after controlling for circulating IL-6. The relationship between insulin sensitivity and circulating zonulin might be mediated through the obesity-related circulating IL-6 increase [13]. In fact, supporting this observation, zonulin gene has been recently reported to coincide with pre-haptoglobin 2 [14], whose promoter is under IL-6 control through STAT3 activation and miR-18a induction [15].

Recent evidence suggests a possible role of gut in obesity. Recent findings emphasize the important symbiotic contributions (in diversity and function) to the human metabolism made by the collection of microbial genomes known as the *microbiome* [16]. Metabolic activities from human gut microbiota correspond to an extra organ equivalent to the liver [17]. Human gut microbiota protects the host against pathogenic microbes by establishing a competitive barrier to their invasion of the mucosal surface, by strengthening the impermeability of the epithelium and by stimulating the development and maintaining the state of alert of the innate and adaptive immune systems [18]. Several studies have shown that high fat diet altered gut microbiote and intestinal permeability promoting metabolic disturbances [19], [20]. According to these studies, the increased inflammation in small intestine associated to obesity and metabolic disturbances may lead to increased in intestinal permeability.

In subjects with glucose intolerance, circulating zonulin was strongly associated with insulin resistance and obesity. Otherwise, in subjects with normal glucose tolerance (NGT), zonulin concentrations were also associated with high levels of uric acid, HbA1c, circulating IL-6 and low HDL-Cholesterol. In conclusion, circulating zonulin might help us to know the contribution of small intestine permeability on glucose intolerance and insulin resistance. Functional ex-vivo studies with recombinant zonulin in mouse intestinal segments have shown that zonulin increased significantly intestinal permeability [14]. The increased zonulin concentration might exert negative effects on intestinal permeability leading to obesity- and insulin resistance-related metabolic disturbances. Further studies are necessary to investigate the possible contribution of zonulin-associated loss of intestinal barrier function on these metabolic disturbances.
